# The eEgg: Evaluation of a New Device to Measure Pain

**DOI:** 10.3389/fphys.2022.832172

**Published:** 2022-03-28

**Authors:** Dshamilja M. Böing-Meßing, Fabian Tomschi, Thomas Cegla, Thomas Hilberg

**Affiliations:** ^1^ Department of Sports Medicine, University of Wuppertal, Wuppertal, Germany; ^2^ Department of Pain Medicine, Helios Klinikum Wuppertal, Wuppertal, Germany

**Keywords:** handgrip, pain, intermodal comparison, dynamometer, clinical applicability, cross-modality matching

## Abstract

**Aim:** The aim of this study was to evaluate whether pain stimuli can be measured validly and reliably by the eEgg (electronic Egg), a new device to measure pain intensity, in comparison to the hand dynamometer.

**Methods:** This study consists of screening and diagnostic tests conforming to the standard criterion of handgrip strength measurement. Fifty healthy participants (25 women, 25 men; age, 39.1 ± 13.7 years) participated in this study. The approach of intermodal comparison was used to transfer different degrees of pain sensations into measurable handgrip strength values. This included an intensity comparison of 10-100% of the subjective maximum handgrip strength and an application of thermal stimuli of 34-48°C. The eEgg was compared to the numeric rating scale (NRS) as a categorization method regarding the subjective assessment of pain. An online questionnaire was distributed to test the evaluation of the product’s features.

**Results:** Regarding the experiment’s validity, the handgrip strength values showed significant (*p* < 0.05) positive correlations between the eEgg and the hand dynamometer (intensities: r=0.328 to r=0.550; thermal stimuli: r=0.353 to r=0.614). The reliability results showed good to very good correlations (*p* < 0.05) in the calculated ICC (intraclass correlation coefficient) values between the individual measurement devices: eEgg intensities: ICC=0.621 to 0.851; thermal stimuli: ICC=0.487 to 0.776 and hand dynamometer intensities: ICC= 0.789 to 0.974; thermal stimuli: ICC=0.716 to 0.910.

**Conclusion:** The new eEgg device shows strong correlations with the hand dynamometer. The central limitation focuses on the obligatory use of an arbitrary unit (AU) for the eEgg. The results of the study indicate that this device can be used in medical and therapeutic practice in the future.

## Introduction

The measurement of pain is essential for pain management, which is of major importance for research on and therapy of acute and chronic pain. Especially the transfer of the highly individual pain perception to numerical values presents a measurement difficulty in clinical practice and the measurement of pain intensity and pain perception is a particular challenge (Fillingim et al., 2016; Manworren and Stinson, 2016; Darbari and Brandow, 2017). Most methods for pain measurement are relying on its subjective evaluation, e.g., the numeric rating scale (NRS) or the visual rating scale (VAS) (Bijur et al., 2003; Williamson and Hoggart, 2005). Further methods, such as pressure pain or heat thresholds, can be used by applying experimental pain stimuli to measure pain sensitivity. In the case of pressure pain thresholds, mechanical stimuli are usually applied *via* pressure algometers by which pressure is applied to a certain body site of the individual with increasing pressure. The individual then states verbally when the pressure exerted becomes painful for the first time. Thus, pain thresholds can be determined, and the pressure applied can be read out in Newton and pain can be measured in a semi-objective manner (Hansen, 1997; Krüger et al., 2021; Luedi et al., 2021). Another method that can be used to evaluate pain is to ask the individual to translate his/her current pain state into another sensory modality, e.g., handgrip strength (Rowbotham, 2001). This method of pain assessment is founded on the theory of intermodal comparison. This theory describes by which means the intensity of pain (usually point prevalence) can be translated into another sensory modality. Subjectively perceived pain is thereby expressed *via* the hand force exerted on a separate device, e.g., the hand dynamometer (HD) (Schandry and Beltz, 2016). Thus, the subjectively experienced pain can be read out in Newton and the individual’s pain is measured semi-objectively. This principle is also known as cross-modality matching (Seidel et al., 1988). Hence, diverse types of pain can be evaluated using the intermodal comparison, e.g., headache, post-operative pain etc., making this method usable in different contexts. The HD is the standard device for the measurement of handgrip strength showing high reliability and validity (Güclüöver et al., 2015; Paramasivan et al., 2019). The HD was also used in previous research that employed the intermodal comparison principle to evaluate pain (Gracely et al., 1978).

Based on these considerations new devices are to be developed to evaluate the individual’s current pain state by asking the individuals to express their current pain state *via* pressing an external device. To the best of our knowledge, the above-mentioned procedure is today only employed by using a HD. The HD was developed to measure handgrip strength and not for the purpose of the intermodal comparison. Hence, the pain measurement device “eEgg” was developed to make use of this procedure and to allow a semi-objective measurement of pain. The eEgg is presented in the study presented herein for the first time and the study aimed to evaluate whether experimental pain stimuli can be measured validly and reliably using the eEgg. Based on these considerations, the following hypotheses were stated: 1) the eEgg is a reliable and valid device to measure experimental pain stimuli of different intensities. 2) The eEgg and the NRS are comparable according to semi-objective and subjective criteria regarding experimental pain stimuli. 3) The eEgg is a feasible and well accepted tool to measure pain in practical use.

## Methods

### Ethics

The study and the used protocols were approved by the ethics committee of the University of Wuppertal at the 16th of May 2019. These protocols are in line with the Declaration of Helsinki. Participants gave written informed consent to participate in the study and were verbally instructed about the procedures conducted in this study.

### Participants

The sample size was calculated a priori *via* G*Power (Version 3.1.9.4) for an assumed moderate correlation between the eEgg and the HD and an alpha-error probability of 0.05 and a power of 0.80 was assumed. This resulted in a sample size of at least 46 subjects. Considering possible dropouts, 50 healthy volunteers participated in the study (Table 1). No participants were excluded from the study. The age distribution was as follows: 20–28 (N = 16/7♂ 9♀), 29–38 (N = 11/6♂ 5♀), 39–48 (N = 8/4♂ 4♀), and 49–65 (N = 15/8♂ 7♀). All participants completed the first experimental test and the online questionnaire. Yet, one participant did not conduct the second experimental test due to personal reasons and two other participants did not finish the online questionnaire.

**TABLE 1:** Anthropometric data of the participants. Data are presented as means ± standard deviation (Range).

Variables mean ± SD (min-max)		
Sex	male (N = 25)	female (N = 25)
Age [years]	39.8 ± 13.9 (23–61)	38.4 ± 13.8 (23–62)
Height [cm]	181.8 ± 8.0 (165–196)	169.2 ± 5.0 (162–183)
Weight [kg]	80.8 ± 10.5 (60–97)	72.0 ± 14.1 (57–103)

Participants were considered eligible for inclusion if they were aged between 18 and 65 years. Participants were excluded if they suffered from any known neurological condition (e.g., multiple sclerosis), or disease that could limit hand function (e.g., carpal tunnel syndrome), reported any acute or chronic pain, or used pain medication regularly. In- and exclusion criteria regarding pain were assessed by the German PainDETECT^©^ questionnaire (Freynhagen et al., 2006).

### Study Design

The aim of the present study was to test the reliability (*via* a test-restest design) and validity (*via* the correlation to the HD as a standard) of the eEgg. Participants were asked to transfer the perceived pain sensation into handgrip strength applied to the eEgg and the HD, respectively. It was further elaborated, whether the semi-objective results of the eEgg can be compared with the subjective data from the NRS. Lastly, the acceptance and the evaluation of the product´s characteristics of the eEgg (e.g., egg size, egg hardness, and egg color) were assessed subsequently using an online questionnaire.

**FIGURE 1:**
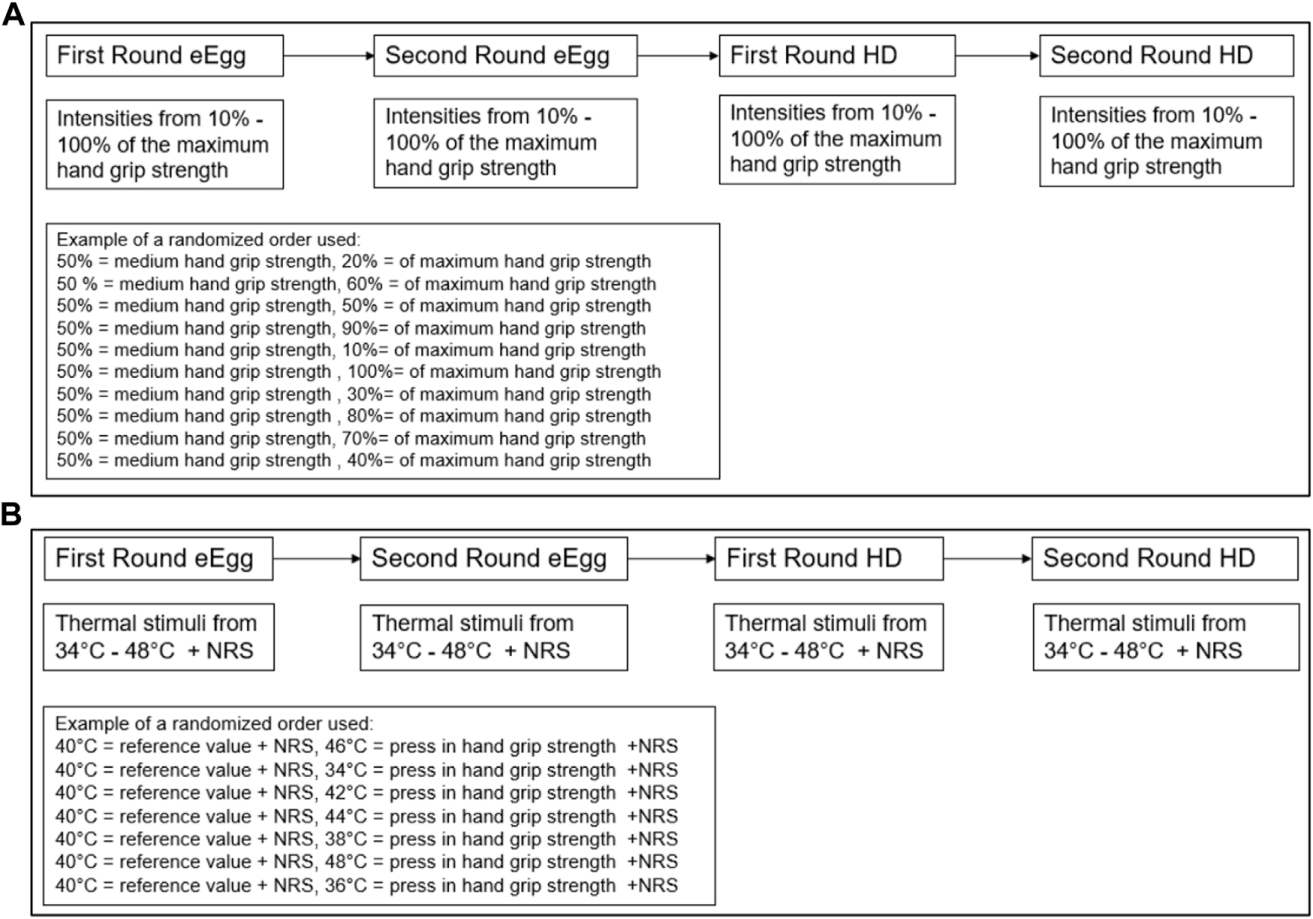
Study design of the first **(A)** and second **(B)** experimental tests. The order of the devices used (eEgg or hand dynamometer) was randomized. *eEgg, electronic egg; HD, hand dynamometer.*

**FIGURE 2:**
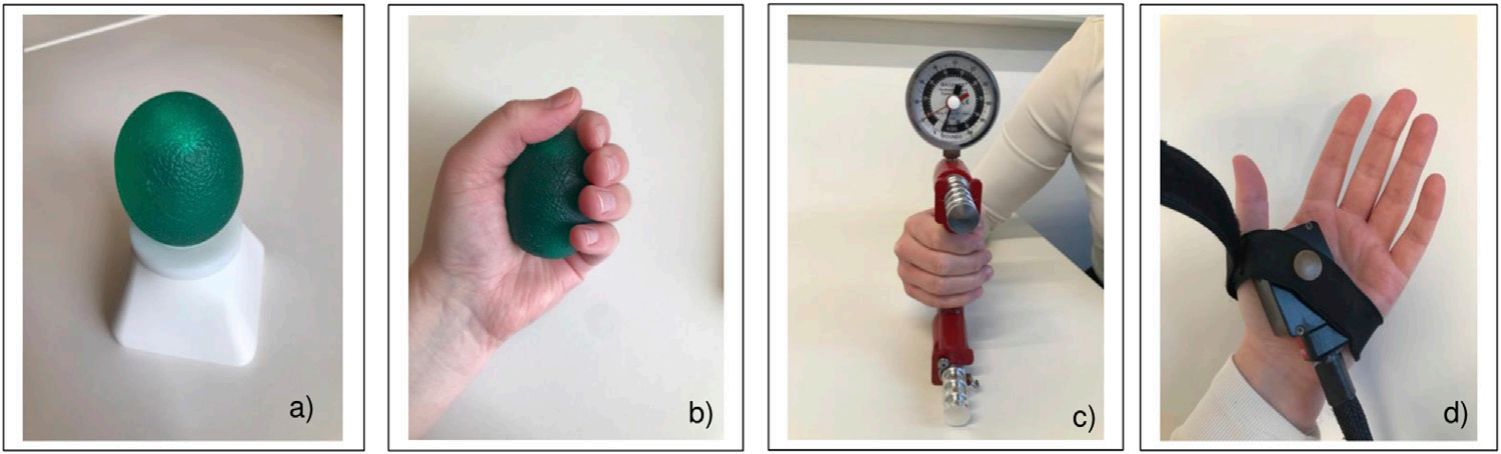
**(A)** The eEgg (electronic egg) in its docking station. **(B)** The position of the eEgg during its application with pressure sensors inside. **(C)** The hand dynamometer and its position during its application. **(D)** The application of the thermal stimuli using the Thermo Sensory Analyzer positioned at the lower part of the palm.

To do so, the study design consisted of three parts. In the first experimental test (see Figure 1A) the participants were asked to express 10–100% of their maximum handgrip strength in intervals in steps of 10% in a randomized order for both devices, i.e., with the eEgg (Figures 2A,B) and the lite hydraulic hand dynamometer 12-0241 (Baseline^®^ USA; Figure 2C). Reference benchmarks were provided verbally with 100% of the handgrip strength being considered to be the maximum handgrip force that could possibly be applied to the eEgg and HD, respectively. Further, 50% of the handgrip strength was considered to resemble a firm handshake, and 0% was instructed as not pressing the devices but only holding them. The participants pressed a reference value of 50% every time before a new intensity was pressed. This test was conducted two times (first and second round) in a test-retest-design.

In the second experimental test (see Figure 1B), thermal stimuli of 34–48°C were applied. The participants expressed the perceived heat intensity as handgrip strength for each trial twice (first and second round) in a test-retest-design. Additionally, they indicated their sensation of pain resulting from the heat stimuli *via* a NRS. The participants pressed a reference value (40°C) every time before reacting to the next heat stimulus and between every thermal stimulus.

The order of the devices used in the first and second experimental tests was randomized. Randomization was conducted using a randomization generator (https://www.random.org/lists). The subjects were randomly assigned to the different groups. In the third part of this study, participants were asked to fill out an online questionnaire to evaluate the eEgg characteristics, which can be found in Supplementary Material S4.

### Material/Measurement

The eEgg (see Figures 2A,B) employs contact sensors to measure the pressure on the surface material. The three-dimensional pressure forces are recorded by several sensors, which are located in a sensor sleeve. This sensor sleeve has an elongated shape and is located in the centre of the eEgg. The sensors provide signals that are then bundled into a sum signal. This sum signal consists of all the average values of the individual sensors. The data from the sum signal are then transferred to device-related software (eEGG_V1.4) *via* Bluetooth and displayed in an Excel file. Via their graphic representation, the values of the respective manual pressure measurement of each run can be read out.

The values expressed by the eEgg do not possess a known physical measurement unit and the values are expressed in an arbitrary numerical unit (AU) based on a positive metric scale with higher values indicting higher applied pressure. The measured pressure is related to the individual base pressure. This basic pressure is created by the effect of the material on the sensors and the position of the eEgg in the hand. Meaning that different basic pressures can have an effect on the eEgg for each measurement (Bromm and Rottmann, 2018). Yet, this is later subtracted from the pressed values in the evaluation by the software.

In the second experimental test, thermal stimuli were applied to the heel of the hand (see Figure 2D) to induce a heat sensation using the Thermo Sensory Analyzer 2001-I (TSA, Medoc, Ramat Yishai, Israel). The baseline temperature was set to 32°C and lasted for 15 s and the temperature was increased by 1°C/s up to the final temperatures, e.g., 48°C, which lasted for 8 s.

After each trial, the pain perception was assessed using the NRS, which ranges from 0 (no pain)–100 (max. pain). The NRS represents a validated pain assessment tool (Williamson and Hoggart, 2005; Hjermstad et al., 2011; Alghadir et al., 2018).

Furthermore as the third part, the study design included an online questionnaire with 29 questions which were specifically designed to evaluate the acceptance and usability of the eEgg. The questions were formulated regarding the acceptance and the evaluation of the product´s features of the eEgg in practical use. Besides, questions concerning the comparison between the eEgg and the HD and regarding the future use of the eEgg were asked. The duration of the processing the questionnaire was approximately 10–15 min. The answers were designed in a Likert scale: 1 = *strongly disagree*; 2 = *disagree*; 3 = *neither agree or disagree*; 4 = *agree;* and 5 = *strongly agree* (see Supplementary Material S4).

### Statistical Analysis

For statistical analysis, SPSS for Windows (Version 27.0; SPSS Inc., Chicago, IL, USA) was used. The Shapiro–Wilk test was used to test the normal distribution indicating mainly non-normal distributed data. Therefore, non-parametric tests were employed to conduct the statistical calculations.

Reliability was assessed in the first and second experimental test during the test-retest procedure by examining the intraclass correlation (ICC) values for the eEgg and HD, respectively. The ICC was interpreted according to Koo and Li, 2016. The ICC can be interpreted as: <0.50 “poor”, 0.50–0.75 “fair”, 0.75–0.90 “good,” and 0.90–1.0 “excellent” (Koo and Li, 2016). Further, the standard error of measurement (SEM) was calculated using the following formula: $SEM=SD\sqrt{1-r}\;$ (Eliasziw et al., 1994).

The validity was determined by calculating the correlation coefficient between the eEgg and the HD in the first and second experimental test according to the rank correlation by Spearman. The correlation coefficient was interpreted according to (Cohen et al., 1988) (r = 0.10: low or weak correlation; r = 0.30: medium or moderate correlation; r = 0.50: large or strong correlation).

Handgrip strength values measured with the eEgg and HD, as well as NRS values, in response to thermal stimuli of the second experimental test are presented as means (± standard deviation). NRS values resulting from the thermal stimuli in the second experimental test were compared using the Mann-Whitney-U test. Handgrip values of the eEgg and HD of adjacent temperatures were compared using the Wilcoxon test within one device. Differences were considered to be significant with a *p*-value of <0.05. The program Limesurvey was used for the online questionnaire and the results are presented as percentages.

## Results

### Results of the First Experimental Test—Intensity

**FIGURE 3:**
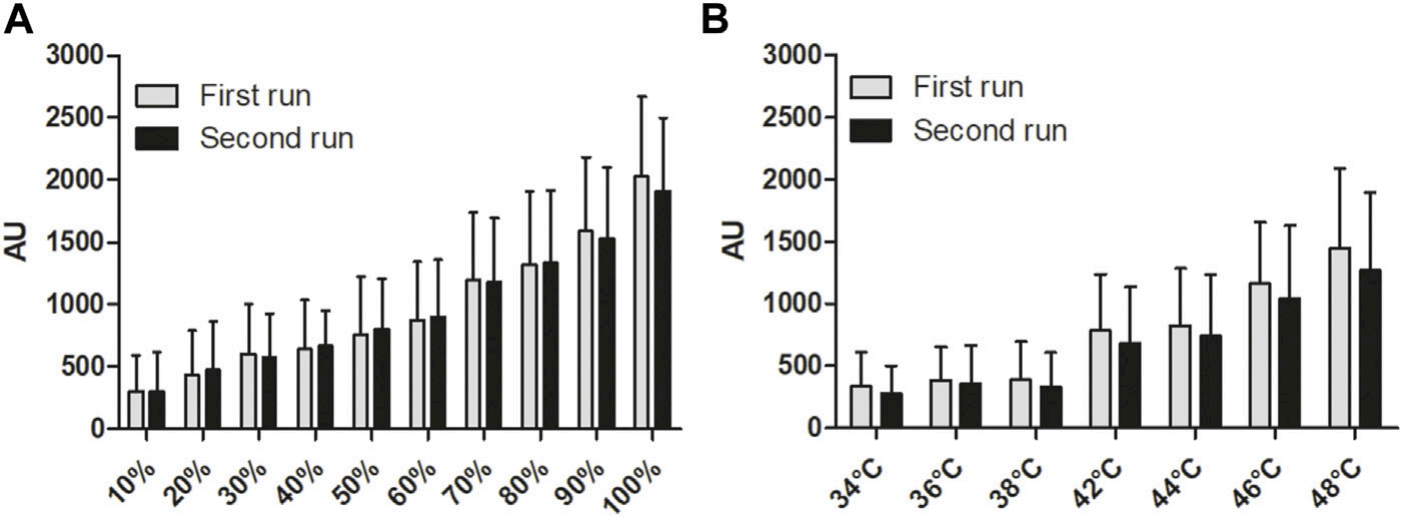
Test-retest results of the eEgg. Handgrip strength values from the eEgg (AU) according to **(A)** different intensities and **(B)** different temperatures of the first and second run, respectively. Data are presented as mean values ± standard deviation. *eEgg, electronic egg.*

With respect to the first reliability testing, data of the first and second run of the test-retest procedure of the eEgg are illustrated in Figure 3A and data of the eEgg and HD are presented in Supplementary Material S1. ICC values of the HD are higher than the eEgg’s ICC values regarding the different intensities (see Table 2 left). The ICC values of the HD range from 0.789 to 0.974, whereas the ICC values of the eEgg range from 0.621 to 0.851. The SEM values of the eEgg ranged from 129.0 to 308.7 and of the HD from 2.0 to 3.0 (see Table 2 left).

**TABLE 2:** Test-retest-reliability—ICC and SEM values: average measurements, intensity and thermal stimuli.

Intensity	First Experimental Test–Intensity						Second Experimental Test–Temperature (°C)						
	eEgg			HD			Temperature	eEgg			HD		
	ICC	p	SEM	ICC	p	SEM		ICC	p	SEM	ICC	p	SEM
10%	0.777	<0.001	129.0	0.857	<0.001	2.1	34°C	0.532	0.004	142.5	0.834	<0.001	1.6
20%	0.748	<0.001	168.0	0.789	<0.001	2.6	36°C	0.667	<0.001	144.4	0.716	<0.001	2.2
30%	0.772	<0.001	163.9	0.902	<0.001	2.1	38°C	0.487	0.011	170.3	0.854	<0.001	1.5
40%	0.773	<0.001	166.9	0.877	<0.001	2.3	42°C	0.752	<0.001	204.0	0.820	<0.001	2.6
50%	0.721	<0.001	203.6	0.899	<0.001	2.4	44°C	0.725	<0.001	222.2	0.910	<0.001	2.3
60%	0.778	<0.001	196.0	0.875	<0.001	2.8	46°C	0.728	<0.001	252.2	0.854	<0.001	2.9
70%	0.741	<0.001	240.8	0.904	<0.001	2.4	48°C	0.776	<0.001	273.7	0.827	<0.001	3.7
80%	0.621	0.001	308.7	0.926	<0.001	2.9	—	—	—	—	—	—	—
90%	0.751	<0.001	260.1	0.927	<0.001	3.0	—	—	—	—	—	—	—
100%	0.851	<0.001	222.2	0.974	<0.001	2.0	—	—	—	—	—	—	—

HD, hand dynamometer; ICC, intraclass coefficient; SEM, standard error of measurement; *p*-values are considered significant with *p* < 0.05.

Regarding validity testing, the values of the eEgg and the values of the HD show a positive correlation (range: r = 0.328 - r = 0.550) with respect to the different intensities representing a medium to strong effect when comparing the handgrip strength values from the intensity run of the eEgg and of the HD (see Table 3).

**TABLE 3:** Correlation values of the handgrip strength values of the eEgg and the hand dynamometer.

Correlations Between eEgg and Hand Dynamometer					
First experimental test—intensity			Second experimental test - temperature		
Intensity	r	p	Temperature	r	p
10%	0.550	<0.001	34°C	0.478	<0.001
20%	0.328	0.020	36°C	0.353	0.013
30%	0.454	<0.001	38°C	0.557	<0.001
40%	0.494	<0.001	42°C	0.571	<0.001
50%	0.398	0.004	44°C	0.460	<0.001
60%	0.452	<0.001	46°C	0.449	0.001
70%	0.470	<0.001	48°C	0.614	<0.001
80%	0.372	0.008	—	—	—
90%	0.396	0.004	—	—	—
100%	0.368	0.008	—	—	—

r = correlations coefficient according to Spearman; *p*-values are considered significant with *p* < 0.05.

**FIGURE 4:**
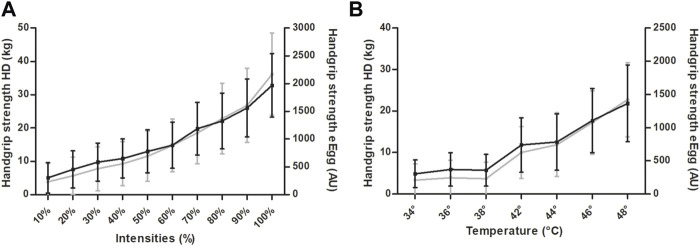
Mean comparison of the handgrip strength values of the eEgg and the hand dynamometer (HD). **(A)** In response to intensities ranging from 10 to 100% of the maximum handgrip strength. **(B)** In response to thermal stimuli ranging from 34 to 48°C. Data are presented as mean ± standard deviation. Grey colour indicates hand dynamometer (HD); Black colour indicates eEgg. *eEgg, electronic egg.*

The results of the first experimental test show a similar increase of mean values of both the HD and the eEgg. The average maximum handgrip strength measured by the eEgg (AU) resulted in a mean of 1969.0 (±575.7). The average maximum handgrip strength of the HD (kg) resulted in a mean of 36.1 (±12.3). The handgrip strength values increased with growing intensities (see Figure 4A and Supplementary Material S1 for data presentation).

### Results of the Second Experimental Test - Temperature

Regarding the second reliability testing, data of the first and second run of the test-retest procedure of the eEgg are illustrated in Figure 3B and data of the eEgg and HD are presented in Supplementary Material S2. The ICC values at different temperatures using the eEgg show a range from 0.487 to 0.776. The ICC values using the HD range from 0.716 to 0.910 (see Table 2 right). In the second experimental test, the SEM values of the eEgg ranged from 142.5 to 273.7 and of the HD from 1.5 to 3.7 (see Table 2 right).

With respect to validity testing, the correlations of the handgrip strength values between the eEgg and the HD after thermal stimuli show significant positive correlations ranging from r = 0.353 to r = 0.614 indicating a medium to strong correlation (see Table 3).

The pain perception assessed by applying heat stimuli showed that the handgrip strength values of both devices follow the same trend and increased similarly (see Figure 4B and Supplementary Material S2 for data presentation). Statistical analyses revealed that significant differences in the handgrip strengths between adjacent temperatures were observed between 34 and 36°C, 38 and 42°C, 44 and 46°C, as well as 46 and 48°C in the eEgg and between 34 and 36°C, 38 and 42°C, 42 and 44°C, 44 and 46°C, as well as 46 and 48°C in the HD.

**FIGURE 5:**
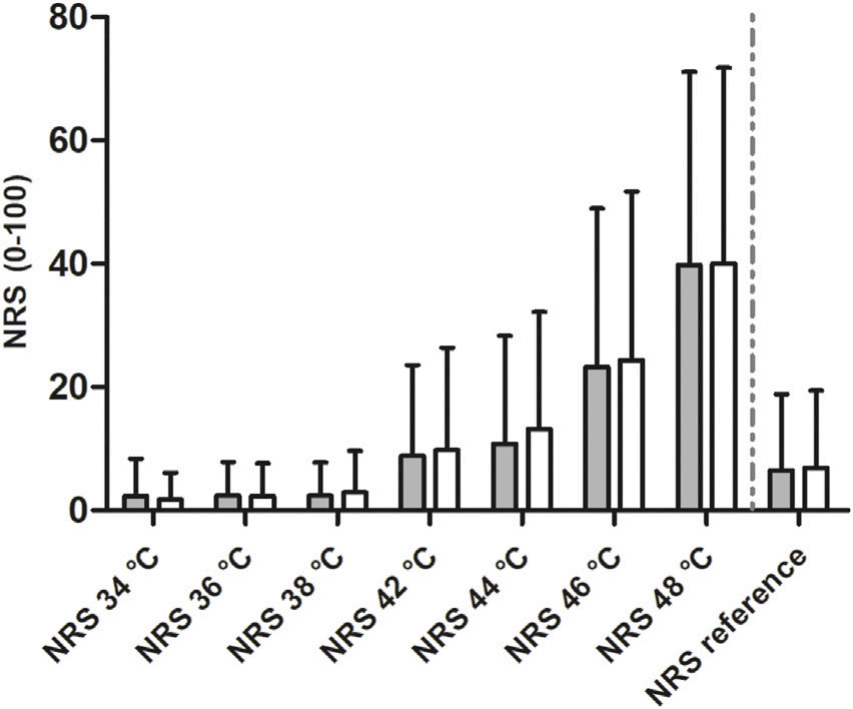
Comparison of the NRS values resulting from thermal stimuli during the second experimental test employing the hand dynamometer (grey filling) and the eEgg (white filling), respectively. Data are presented as means ± standard deviation. No significant difference is observed between the NRS values. *NRS, Numerical Rating Scale; eEgg, electronic egg.*

When comparing the NRS values resulting from the heat stimuli in the run using the eEgg and the HD, respectively, results show that the NRS values do not differ between both runs, with *p* > 0.05 for all temperatures (see Figure 5 and Supplementary Material S3 for data presentation).

### Results of the Online Questionnaire

The participants rated their first and final impression of the eEgg in grades from 1 (best) to 6 (worst). The average grade overall was two. Further results are presented in Table 4.

**TABLE 4:** Selected results of the online questionnaire N = 48.

	1 = strongly Disagree (%)	2 = disagree (%)	3 = neither Agree or Disagree (%)	4 = agree (%)	5 = strongly Agree (%)
HD more comfortable	58.3	31.3	4.2	4.2	2.1
eEgg measures imprecisely	47.9	29.2	10.4	10.4	2.1
eEgg measures more precisely	0.0	4.2	18.8	39.6	37.5
Material comfortable	6.3	0.0	14.6	41.7	37.5
Position comfortable	4.2	4.2	14.6	35.4	41.7
Shape comfortable	2.1	2.1	4.2	37.5	54.2
Handling comfortable	2.1	2.1	12.5	41.7	41.7
Preference eEgg	2.1	8.3	12.5	39.6	37.5
NRS easier to express	35.4	33.3	18.8	10.4	2.1
eEgg easier to express	2.1	8.3	25.0	43.8	20.8
Future use	4.2	4.2	27.1	45.8	18.8

NRS, numerical rating scale; HD, hand dynamometer.

## Discussion

The hypotheses stated in the context of this study were threefold and summarizing the results regarding these hypotheses can be stated as follows: 1) it can be concluded that the eEgg presents different handgrip strengths, indicated as different percentages of the maximum handgrip strength and indicated as handgrip strength in response to different experimental heat stimuli, in a reliable manner with fair to good ICC values. With respect to validity testing, the study results show that the eEgg shows medium to large correlations with the HD. 2) it can be concluded that NRS responses, as a subjective measure, and handgrip responses using the eEgg, as a semi-objective measure, is similar. 3) it was shown that the eEgg is perceived as a pleasant and feasible device to measure pain in a semi-objective manner.

More specifically, a test-retest reliability in a fair to good correlation state was observed for the eEgg regarding the first experimental test regarding intensity and a fair to good correlation in the second experimental test regarding temperature. Both results indicate that the eEgg is reliable in a wide spectrum of handgrip intensities (10–100% of maximum handgrip strength) and, more importantly, in wide spectrum of experimental pain stimuli (34–48°C). Therefore, the eEgg seems to be a tool that is able to document differently intensive subjective pain states in a semi-objective manner. However, the handgrip strength values of the eEgg show overall lower ICC values than the ones obtained by the HD which is considered to be the gold standard for handgrip measurements, also in the context of intermodal comparisons (Gracely et al., 1978). The reliability of the values of the HD used in this study are in line with previous studies (Allen and Barnett, 2011; Güçlüöver et al., 2015; Paramasivan et al., 2019).

Further, this study demonstrated that the eEgg measures hand pressure intensities and thermal stimuli intensities in a valid manner compared to the HD as the gold standard. With respect to the first and second experimental tests the handgrip strengths measured correlated positively when using the eEgg and the HD indicating medium to large correlations in the first and second experimental tests. More specifically, this correlation was observed in the entire spectrum used in this study. I.e., significant positive correlations were observed for all intensity tests (10–100% of maximum handgrip strength) and for all temperatures, except for one temperature (36°C). This observation might be attributed to the impact of the ambient and human body temperature on pain sensation might have been an influencing factor (Strigo et al., 2000; Gekle and Singer, 2010; Obermeyer et al., 2017). The handgrip strength values (369.7 AU) pressed at 36°C are slightly higher than the handgrip strength values (357.1 AU) pressed at 38°C, though they are not significantly different. The handgrip strength values of the higher temperature ranges are more clearly distinguishable from each other. The thermal stimuli higher than 42°C were more clearly distinguishable by the participants no matter what device was used as adjacent temperatures showed significant differences. The temperatures of 34, 36, and 38°C are similar to the human body temperature and it is most likely difficult to feel the difference between the respective temperatures. Yet, significant differences in handgrip pressure were observed between the adjacent temperatures 34 and 36°C employing the eEgg indicating a good sensitivity. Yet, no such difference was observed between 36 and 38°C. This result was observed for the HD as well. Each individual possesses a different basic body temperature due to their gender, age, weight, and demographic conditions (Obermeyer et al., 2017). These results underline the difficulty of differentiating the values close to the body temperature by handgrip strength and might be one reason for the lack of a significant correlation between the eEgg and the HD observed at 36°C.

The NRS values represent a subjective pain rating by the individual (Bijur et al., 2003; Alghadir et al., 2018). NRS values recorded in the second experimental test demonstrated an increase with increasing temperatures, which was to be expected. This increase is also observed in the semi-objective values expressed by the Egg and it can be observed that higher subjective pain ratings *via* NRS go along with higher semi-objective values using the eEgg, as well as the HD. For the measurement of pain, the self-report by the patient is of crucial importance. Therefore, the NRS, and also the VAS, are the pain measurement tools, which are usually used in clinical practice (Haefeli and Elfering, 2006; Gélinas, 2016). In addition, in the context of sport sciences, various scales, above all the rate of perceived excretion (RPE) scale, are used to subjectively scale exertion, breathlessness, and fatigue usually during a standardized ergometer test (Williams, 2017). Moreover, various pain scales are also used to evaluate bodily and muscular pain (O’connor et al., 2000). One example is the CR 10 scale, which was initially developed to measure exertion as well as pain, e.g., muscular and/or exertional pain (Borg, 1998). The eEgg presents a new way to measure pain providing numerical readouts, which might also be used in the context of sports science and sport medicine.

In this study, participants of broad age range were included, and the age distribution might be biased towards the younger and older individuals. Yet, the influence of age on pain perception is mostly recognized when obtaining pain thresholds with higher pain thresholds observed in older people, which was not done in this study (Lautenbacher et al., 2017). Participants had to rate their perceived pain sensation on the NRS and employing the respective devices.

The results of the online questionnaire illustrate the participants’ impression of the more precise and more comfortable use of the eEgg in comparison to the HD, which can be used to employ the principle of the intermodal comparison (Gracely et al., 1978). Influencing factors might be the soft surface material and the overall larger contact area of the hand with the eEgg. In contrast to the HD, the flexible material of the eEgg provides a pleasant feedback feeling. This could have led to a better-perceived assessment ability of the participants’ handgrip strength.

Using the eEgg with a connected mobile application presents one development goal which might improve pain management in clinical settings in the future as more and more measurement tools can be employed with apps and possess multiple digital features. Recent studies highlight the potential of these mobile digital apps for more accurate pain management (Zhao et al., 2019; Zheng et al., 2020). Due to the already existing digital data transmission *via* Bluetooth, the eEgg has the prerequisite to be connected to compatible apps and devices in the future.

### Limitations

Pain is a subjective sensation that cannot be measured objectively. Therefore, the measurements made underly some kind of subjective variance that cannot be excluded. Yet, the eEgg provides numerical values that can be read out by clinicians and used to interpret the patient’s pain providing a semi-objective evaluation. However, the major limitation of the eEgg is the missing standardised unit. Instead, an arbitrary unit is employed. Due to the fact that this is the first study presenting results of the eEgg, the obtained results cannot be compared to other studies or data. But, the development and calibration of a standardised measurement unit would allow a more decisive statistical analysis and an even more precise comparison between the eEgg and other devices, such as the HD, and the results presented herein present a starting point for future research and development.

## Conclusion

The evaluation of the eEgg regarding reliability and validity shows that the eEgg is a reliable device to measure pain in a semi-objective manner with fair to good ICC values. Yet, the ICC values of the test-restest reliability testing are lower when using the eEgg compared to the HD. Validity testing by comparing the eEgg to the HD revealed medium to large correlations in both experimental tests conducted. It needs to be mentioned that the eEgg employs an arbitrary unit and the inclusion of a physical unit is suggested. The handling of the eEgg is perceived as more pleasant by the participants compared to the HD. The eEgg can be considered as an usable device for the measurement of pain that can be employed in further clinical and therapeutic settings.

## Data Availability

The data that support the findings of this study are available from the corresponding author, DB-M, upon reasonable request.

## References

[B1] Alghadir A. Anwer S. Iqbal A. Iqbal Z. (2018). Test–retest Reliability, Validity, and Minimum Detectable Change of Visual Analog, Numerical Rating, and Verbal Rating Scales for Measurement of Osteoarthritic Knee Pain. Jpr Vol. 11, 851–856. 10.2147/JPR.S158847 PMC5927184 29731662

[B2] Allen D. Barnett F. (2011). Reliability and Validity of an Electronic Dynamometer for Measuring Grip Strength. Int. J. Ther. Rehabil. 18, 258–264. 10.12968/ijtr.2011.18.5.258

[B3] Banzer W. Bürklein M. (2004). “Funktionsdiagnostik des Bewegungssystems in der Sportmedizin,” in Funktionsdiagnostik des Bewegungssystems in der Sportmedizin. Editors Banzer W. Pfeifer K. Vogt L. (Springer-Verlag Berlin Heidelberg). 10.1007/978-3-642-18626-4

[B4] Bijur P. E. Latimer C. T. Gallagher E. J. (2003). Validation of a Verbally Administered Numerical Rating Scale of Acute Pain for Use in the Emergency Department. Acad. Emerg. Med 10 (4), 390–392. 10.1111/j.1553-2712.2003.tb01355.x 12670856

[B5] Borg G. (1998). Borg's Perceived Exertion and Pain Scales. Hum. kinetics.

[B6] Bromm B. Rottmann F. (2018). Schmerzmessung mit Hilfe des eEGGs.

[B7] Cohen J. Hillsdale N. J. (1988). Statistical Power Analysis for the Behavioral Sciences. 2nd ed. L. Erlbaum Associates. 10.1016/C2013-0-10517-X

[B8] Darbari D. S. Brandow A. M. (2017). Pain-measurement Tools in Sickle Cell Disease: where Are We Now? Hematol. Am. Soc. Hematol. Edu. Program 2017 (1), 534–541. 10.1182/asheducation-2017.1.534 PMC6142608 29222302

[B10] Eliasziw M. Young S. L. Woodbury M. G. Fryday-Field K. (1994). Statistical Methodology for the Concurrent Assessment of Interrater and Intrarater Reliability: Using Goniometric Measurements as an Example. Phys. Ther. 74 (8), 777–788. 10.1093/ptj/74.8.777 8047565

[B11] Fillingim R. B. Loeser J. D. Baron R. Edwards R. R. (2016). Assessment of Chronic Pain: Domains, Methods, and Mechanisms. The J. pain 17 (9 Suppl. l), T10–T20. 10.1016/j.jpain.2015.08.010 27586827 PMC5010652

[B12] Freynhagen R. Baron R. Gockel U. Tölle T. R. (2006). painDETECT: a New Screening Questionnaire to Identify Neuropathic Components in Patients with Back Pain. Curr. Med. Res. Opin. 22 (10), 1911–1920. 10.1185/030079906X132488 17022849

[B13] Gekle M. Singer D. (2010). “Temperaturregulation und Wärmehaushalt,” in Physiologie. Georg Thieme Verlag 8. Editors Pape H. C. Kurtz A. Silbernagl S. (unveränderte Auflage.

[B14] Gélinas C. (2016). Pain Assessment in the Critically Ill Adult: Recent Evidence and New Trends. Intensive Crit. Care Nurs. 34, 1–11. 10.1016/j.iccn.2016.03.001 27067745

[B15] Gracely R. H. McGrath P. Dubner R. (1978). Validity and Sensitivity of Ratio Scales of Sensory and Affective Verbal Pain Descriptors: Manipulation of Affect by Diazepam. Pain 5 (1), 19–29. 10.1016/0304-3959(78)90021-0 673439

[B16] Güçlüöver A. Kutlu M. Ciğerci A. E. Esen H. T. Demirkan E. Erdoğdu M. (2015). Determination the Validity of the New Developed Sport Experts® Hand Grip Dynamometer, Measuring Continuity of Force, and Comparison with Current Takei and Baseline® Dynamometers. The J. Sports Med. Phys. fitness 55 (11), 1318–1321. PMID: 25289714Haefeli,. 25289714

[B9] Haefeli M. Elfering A. (2006). Pain Assessment. Eur. Spine J. 15 (1), S17–S24. 16320034 10.1007/s00586-005-1044-x PMC3454549

[B17] Hansen B. (1997). Through a Glass Darkly: Using Behavior to Assess Pain. Semin. Vet. Med. Surg. Small Anim. 12 (No. 2), 61–74. 10.1016/s1096-2867(97)80003-5 9159063

[B18] Hjermstad M. J. Fayers P. M. Haugen D. F. Caraceni A. Hanks G. W. Loge J. H. (2011). Studies Comparing Numerical Rating Scales, Verbal Rating Scales, and Visual Analogue Scales for Assessment of Pain Intensity in Adults: a Systematic Literature Review. J. Pain Symptom Manage. 41 (6), 1073–1093. 10.1016/j.jpainsymman.2010.08.016 21621130

[B19] Koo T. K. Li M. Y. (2016). A Guideline of Selecting and Reporting Intraclass Correlation Coefficients for Reliability Research. J. chiropractic Med. 15 (2), 155–163. 10.1016/j.jcm.2016.02.012 PMC4913118 27330520

[B20] Krüger S. Herzig M. Hilberg T. (2021). Changes in Pain Profile of Patients with Haemophilia during 1‐year Follow‐up. Haemophilia 27 (5), 783–792. 34390092 10.1111/hae.14380

[B21] Lautenbacher S. Peters J. H. Heesen M. Scheel J. Kunz M. (2017). Age Changes in Pain Perception: a Systematic-Review and Meta-Analysis of Age Effects on Pain and Tolerance Thresholds. Neurosci. Biobehavioral Rev. 75, 104–113. 10.1016/j.neubiorev.2017.01.039 28159611

[B22] Luedi M. M. Schober P. Hammoud B. Andereggen L. Hoenemann C. Doll D. (2021). Preoperative Pressure Pain Threshold Is Associated with Postoperative Pain in Short-Stay Anorectal Surgery: a Prospective Observational Study. Anesth. analgesia 132 (3), 656–662. 10.1213/ane.0000000000005072 PMC7870038 32675636

[B24] Manworren R. C. B. Stinson J. (2016). Pediatric Pain Measurement, Assessment, and Evaluation. Semin. Pediatr. Neurol. 23 (3), 189–200. 10.1016/j.spen.2016.10.001 27989326 PMC5261830

[B25] Nilges P. (2006). “Klinische Schmerzmessung,” in Praktische Schmerztherapie. Editors Baron R. Strumpf M. (Springer-Verlag Berlin Heidelberg).

[B26] O'connor P. J. Murphy R. M. Courson R. W. Ferrara M. S. (2000). Pain Assessment in Journal of Athletic Training Articles 1992-1998: Implications for Improving Research and Practice. J. Athl Train. 35 (2), 151–154. 16558624 PMC1323411

[B27] Obermeyer Z. Samra J. K. Mullainathan S. (2017). Individual Differences in normal Body Temperature: Longitudinal Big Data Analysis of Patient Records. BMJ 359, j5468. Dec 13. 10.1136/bmj.j5468 29237616 PMC5727437

[B23] Paramasivan M. Kiruthigadevi S. Amal K. F. (2019). Test-retest Reliability of Electronic Hand Dynamometer in Healthy Adults. Int. J. Adv. Res. 7, 325–331. 10.21474/IJAR01/9042

[B28] Rowbotham M. C. (2001). What Is a 'clinically Meaningful' Reduction in Pain? Pain 94 (2), 131–132. 10.1016/s0304-3959(01)00371-2 11690725

[B29] Schandry R. Beltz J. (2016). Biologische Psychologie. Weinheim Basel: Beltz.

[B30] Seidel H. Harazin B. Pavlas K. Sroka C. Richter J. Blüthner R. (1988). Isolated and Combined Effects of Prolonged Exposures to Noise and Whole-Body Vibration on Hearing, Vision and Strain. Int. Arch. Occup. Environ. Heath 61, 95–106. 10.1007/bf00381613 3198289

[B31] Strigo I. A. Carli F. Bushnell M. C. (2000). Effect of Ambient Temperature on Human Pain and Temperature Perception. Anesthesiology 92 (3), 699–707. 10.1097/00000542-200003000-00014 10719949

[B32] Williams N. (2017). The Borg Rating of Perceived Exertion (RPE) Scale. Occup. Med. 67 (5), 404–405. 10.1093/occmed/kqx063

[B33] Williamson A. Hoggart B. (2005). Pain: a Review of Three Commonly Used Pain Rating Scales. J. Clin. Nurs. 14 (7), 798–804. 10.1111/j.1365-2702.2005.01121.x 16000093

[B34] Zhao P. Yoo I. Lancey R. Varghese E. (2019). Mobile Applications for Pain Management: an App Analysis for Clinical Usage. BMC Med. Inform. Decis. Mak 19 (1), 106. 10.1186/s12911-019-0827-7 31146739 PMC6543581

[B35] Zheng C. Chen X. Weng L. Guo L. Xu H. Lin M. (2020). Benefits of Mobile Apps for Cancer Pain Management: Systematic Review. JMIR Mhealth Uhealth 8 (1), e17055. 10.2196/17055 32012088 PMC7005688

