# Supplementary material for: The eEgg: Evaluation of a New Device to Measure Pain
Source: Front Physiol. 2022 Mar 28;13:832172. doi: 10.3389/fphys.2022.832172 (PMC8996247; doi:10.3389/fphys.2022.832172)
Supplement: Supplementary file 1 [file Table1.DOCX]

Supplemental material 1:

Handgrip strength values from the eEgg (AU) and from the hand dynamometer (kg) according to different intensities. Data are presented as mean values ± standard deviation (range) from the two runs and presented as one average value.

*SD=standard deviation; HD= Hand dynamometer*

| **Intensity** | **Mean ± SD (Min-Max)** | | | | | |
| --- | --- | --- | --- | --- | --- | --- |
|  | **Intensity of handgrip strength eEgg of the first run** | **Intensity of handgrip strength eEgg of the second run** | **Intensity of handgrip strength eEgg presented as average** | **Intensity of handgrip strength HD of the first run** | **Intensity of handgrip strength HD of the second run** | **Intensity of handgrip strength HD presented as average** |
| **Reference 50%** | 783.2 ± 414.9  (73.0 – 2020.0) | 783.9 ± 420.7  (70.0 – 2023.0) | 783.5 ± 353.3 (203.2-1677.8) | 11.8 ± 8.0  (0.0 – 46.0) | 12.4 ± 8.4  (0.0 – 48.0) | 12.1 ± 7.7 (1.4-41.3) |
| **10%** | 305.9 ± 285.6  (35.0 – 1299.0) | 301.3 ± 318.6  (17.0 – 1778.0) | 303.6 ± 273.2 (43.0-1329.0) | 3.1 ± 4.8  (0.0 – 25.0) | 4.6 ± 6.7  (0.0 – 29.0) | 3.8 ± 5.5 (0.0-27.0) |
| **20%** | 433.6 ± 358.8  (38.0 – 1481.0) | 473.0 ± 389.8  (13.0 – 1585.0) | 453.3 ± 334.6 (34.0-1524.5) | 4.9 ± 5.2  (0.0 – 23.0) | 6.4 ± 6.9  (0.0- 30.0) | 5.7 ± 5.6 (0.0-23.5) |
| **30%** | 601.6 ± 400.9  (61.0-1822.0) | 570.0 ± 359.3  (71.0 – 1401.0) | 585.8 ± 343.2 (83.5-1462.5) | 7.7 ± 6.9  (0.0 – 36.0) | 7.8 ± 6.9  (0.0. – 36.0) | 7.7 ± 6.6 (0.0-36.0) |
| **40%** | 641.5 ± 395.5  (89.0 – 1815.0) | 660.9 ± 281.1  (125.0 – 1725.0) | 651.2 ± 350.2 (152.0-1584.5) | 8.7 ± 6.6  (0.0 – 28.0) | 10.0 ± 7.1  (0.0 – 30.0) | 9.3 ± 6.5 (0.0-29.0) |
| **50%** | 759.5 ± 463.8  (77.0 – 1996.0) | 799.9 ± 407.2  (189.0 – 1893.0) | 779.7 ± 385.5 (177.5-1659.5) | 11.3 ± 7.9  (0.0 – 43.0) | 11.8 ± 7.9  (0.0 – 42.0) | 11.5 ± 7.5 (0.0-42.5) |
| **60%** | 878.5 ± 466.1  (125.0 – 2060.0) | 903.9 ± 454.5  (263.0 – 1990.0) | 891.2 ± 416.0 (206.5-1811.0) | 14.2 ± 8.6  (0.0 – 44.0) | 15.3 ± 8.2  (3.0 – 41.0) | 14.8 ± 7.9 (1.5-42.5) |
| **70%** | 1196.5 ± 544.8 (258.0 – 2415.0) | 1177.5 ± 518.2  (369.0 – 2766.0) | 1186.9 ± 473.2 (460.5-2475.5) | 18.1 ± 9.6  (0.0 – 43.0) | 18.9 ± 9.7  (5.0 – 43.0) | 18.5 ± 9.2 (5.5-42.5) |
| **80%** | 1316.3 ± 596.1  (186.0 – 2529.0) | 1336.3 ± 583.4  (452.0 – 2893.0) | 1326.3 ± 501.5 (474.5-2539.0) | 22.7 ± 11.5  (5.0 – 48.0) | 23.1 ± 10.4  (7.0 – 44.0) | 22.9 ± 10.6 (7.0-44.5) |
| **90%** | 1591.6 ± 594.3  (451.0 – 2888.0) | 1529.3 ± 571.5  (688.0 – 2913.0) | 1560.5 ± 521.3 (648.5-2870.0) | 27.1 ± 12.0  (9.0 – 63.0) | 26.5 ± 11.01  (9.0 – 49.0) | 26.8 ± 11.1 (11.0-56.0) |
| **100%** | 2026.3 ± 643.2  (735.0 – 2911.0) | 1911.7± 586.5  (855.0 – 2904.0) | 1969.0 ± 575.7 (938.0-2892.5) | 36.7 ± 12.4  (16.0 – 71.0) | 35.6 ± 12.6  (15.0 – 68.0) | 36.1 ± 12.3 (18.0-69.5) |
