# Supplementary material for: The eEgg: Evaluation of a New Device to Measure Pain
Source: Front Physiol. 2022 Mar 28;13:832172. doi: 10.3389/fphys.2022.832172 (PMC8996247; doi:10.3389/fphys.2022.832172)
Supplement: Supplementary file 2 [file Table2.DOCX]

Supplemental material 2:

Handgrip strength values from the eEgg (AU) and from the hand dynamometer (kg) according to thermal stimuli. Data are presented as mean values ± standard deviation (range) from the two runs and presented as one average value.

SD=standard deviation; NRS=Numerical Rating Scale; HD= Hand dynamometer

| **Temperature** | **Mean ± SD (Min-Max)** | | | | | |
| --- | --- | --- | --- | --- | --- | --- |
|  | **Intensity of handgrip strength eEgg of the first run** | **Intensity of handgrip strength eEgg of the second run** | **Intensity of handgrip strength eEgg presented as average** | **Intensity of handgrip strength HD of the first run** | **Intensity of handgrip strength HD of the second run** | **Intensity of handgrip strength HD presented as average** |
| **Reference 40°C** | 738.4 ± 417.0  (77.0 – 1946.0) | 636.8 ± 419.0  (67.0 – 1857.0) | 689.1 ± 347.9  (132.6-1507.4) | 10.4 ± 7.3  (0.0 – 38.0) | 9.9 ± 6.7  (0.0 – 33.0) | 10.1 ± 6.3  (0.0-28.6) |
| **34°C** | 336.7 ± 276.2  (79.0 – 1120.0) | 273.1 ± 225.7  (55.0 – 988.0) | 303.9 ± 208.3  (69.0-928.5) | 3.4 ± 4.6  (0.0 – 19.0) | 3.3 ± 3.7  (0.0 – 14.0) | 3.3 ±3.9  (0.0-15.0) |
| **36°C** | 384.6 ± 270.2  (76.0 – 1227.0) | 358.5 ± 308.4  (47.0 – 1323.0) | 369.7 ± 250.3  (77.0-1104.5) | 4.2 ± 5.4  (0.0 – 22.0) | 3.7 ± 3.9  (0.0 – 16.0) | 3.9 ± 4.1  (0.0-16.5) |
| **38°C** | 388.6 ± 304.3  (84.0 – 1601.0) | 329.4 ± 279.0  (67.0 – 1207.0) | 357.1 ± 237.7  (84.5-1084.0) | 3.5 ± 4.3  (0.0 – 14.0) | 3.9 ± 4.3  (0.0 – 20.0) | 3.7 ± 4.0  (0.0-16.5) |
| **42°C** | 784.1 ± 453.0  (105.0 – 2114.0) | 680.5 ± 456.1  (75.0 – 1630.0) | 737.7 ± 409.7  (123.5-1808.0) | 10.5 ± 6.9  (0.0 – 28.0) | 9.5 ± 6.6  (0.0 – 30.0) | 10.0 ± 6.2  (0.0-25.5) |
| **44°C** | 825.1 ± 461.2  (130.0 – 2016.0) | 738.4 ± 499.0  (95.0 – 2212.0) | 781.6 ± 423.7  (168.0-1705.0) | 11.8 ± 8.0  (0.0 – 36.0) | 12.0 ± 8.0  (0.0 – 34.0) | 11.9 ± 7.6  (0.0-35.0) |
| **46°C** | 1163.9 ± 495.4  (235.0 – 2296.0) | 1039.0 ± 591.4  (236.0 – 2598.0) | 1105.2 ± 483.6 (240.0-2432.5) | 18.0 ± 7.6  (5.0 – 36.0) | 16.4 ± 8.6  (3.0 – 38.0) | 17.2 ± 7.6  (4.5-37.0) |
| **48°C** | 1447.9 ± 642.5  (449.0 – 2898.0) | 1269.6 ± 628.5  (242.0 – 2883.0) | 1365.0 ± 578.2 (349.5-2727.5) | 22.8 ± 9.7  (5.0 – 42.0) | 22.6 ± 9.6  (4.0 – 45.0) | 22.7 ± 8.9  (5.0-43.5) |
