# Supplementary material for: The eEgg: Evaluation of a New Device to Measure Pain
Source: Front Physiol. 2022 Mar 28;13:832172. doi: 10.3389/fphys.2022.832172 (PMC8996247; doi:10.3389/fphys.2022.832172)
Supplement: Supplementary file 3 [file Table3.DOCX]

Supplemental material 3:

Associated pain sensations on the NRS resulting from thermal stimuli. Data are presented as mean values ± standard deviation (range) from the averages of two runs. No significant difference (p>0.05) was observed.

| **Temperature** | **Mean ± SD (Min-Max)** | |
| --- | --- | --- |
|  | **NRS-values eEgg** | **NRS-values HD** |
| **Reference 40°C** | 6.9 ± 11.8 (0.0-43.1) | 6.5 ± 11.5 (0.0-37.1) |
| **34°C** | 1.8 ± 4.1  (0.0-17.5) | 2.3 ± 5.6  (0.0-25.0) |
| **36°C** | 2.3 ± 6.3  (0.0-22.5) | 2.3 ± 5.3  (0.0-20.0) |
| **38°C** | 2.9 ± 6.3  (0.0-25.0) | 2.3 ± 5.0  (0.0-20.0) |
| **42°C** | 9.8 ± 16.3  (0.0-67.5) | 8.8 ± 14.2 (0.0-50.0) |
| **44°C** | 13.2 ± 18.4 (0.0-65.0) | 10.7 ± 16.4 (0.0-67.5) |
| **46°C** | 24.4 ± 26.5 (0.0-80.0) | 23.2 ± 25.4 (0.0-75.0) |
| **48°C** | 40.0 ± 31.3 (0.0-100.0) | 39.7 ± 31.2 (0.0-100.0) |
