# Supplementary material for: The eEgg: Evaluation of a New Device to Measure Pain
Source: Front Physiol. 2022 Mar 28;13:832172. doi: 10.3389/fphys.2022.832172 (PMC8996247; doi:10.3389/fphys.2022.832172)
Supplement: Supplementary file 4 [file Table4.DOCX]

**Supplementary material 4 - Online questionnaire eEgg**

1. My first impression of the eEgg (in school grades 1-6)

1.a For the eEgg to be used to measure pain, in my opinion orange is an appropriate color.

1.b For the eEgg to be used to measure pain, in my opinion green is an appropriate color.

1.c For the eEgg to be used to measure pain, in my opinion black is an appropriate color.

2. The color of the eEgg is relevant to me.

3. The material of the eEgg feels comfortable on the skin.

4. The position of the eEgg in the hand is pleasant.

5. The shape of the eEgg pleasant.

6. The handling of the eEgg is pleasant.

7. The size of the eEgg is pleasant.

8. The size of the eEgg is too big for me to press.

9. The size of the eEgg is too small for me to press.

10. The degree of hardness of the green eEgg feels pleasant.

10.a The hardness of the black eEgg feels comfortable for me.

10.b The hardness of the eEgg is relevant to me.

11. The feeling of pressing the eEgg is pleasant.

12. The hardness of the black eEgg is too soft.

12.a. The hardness of the orange eEgg is too hard.

13. The eEgg appears to be of high quality.

14. It is easier for me to express the different intensities of pain with the eEgg than with the numeric rating scale.

15. The hand dynamometer is more comfortable to hold than the eEgg.

16. With the eEgg, my hand pressure cannot be measured as precisely as with the hand dynamometer.

17. With the eEgg, my hand pressure can be measured even more precisely than with the hand dynamometer.

18. To express pain intensity I would prefer the eEgg to the numeric rating scale.

19. Pain intensity can be expressed well with the help of the eEgg.

20. It is difficult for me to adequately translate pain intensity to my handgrip strength.

20a. It is easier to express the pain intensity with the numeric rating scale than with the eEgg.

21. The nature of the material under pressure is too soft to express the pain intensity.

22. It is difficult for me to express the mild pain intensity of 20% using the eEgg.

23. It is difficult for me to express the high pain intensity of 100% using the eEgg.

24. The nature of the material under pressure is too hard to express the pain intensity.

25. I would like to use the eEgg in the future to express my pain intensity.

26. I do not consider the future use of the eEgg to be a problem.

27. I would recommend the use of the eEgg to others.

28. I consider the future use of the eEgg to be questionable.

29. My final overall impression of the eEgg (school grade 1-6)
